# Free Choice of Herbalist

**Published:** 1913-11-15

**Authors:** 


### THE NON-PANEL MOVEMENT.

### Free Choice of Herbalists.

When in January last the British Medical Association confirmed its previously expressed opinion of the conditions of service under the Insurance Act as "derogatory to the profession and against the public interest"—and confirmed it by a resolution passed at a representative meeting of delegates with only one dissentient—it was announcing a view which subsequent events have fully justified. The Association is at present, however, in the unsatisfactory position of finding a majority of its members on the panels; and as any future steps will presumably depend on the views of that majority, the Association must now unfortunately be regarded as a pro-panel body; for its members have accepted the conditions of service despite the verdict of the representative body. A special representative meeting is to be held early in December, and it will be interesting to see whether there will be a repetition of the attempt made in July to rescind the "derogatory" resolution, and if so with what result. Until such rescinding takes place, the anomalous position of the Association is that its theory and practice are opposed, and all logic and consistency are clearly on the side of the non-panel members, who, being in conformity with the official opinion, have declined service under the Act.

### The B.M.A. and Non-Panel Men.

Further evidence that the Association may expect difficulty in retaining non-panel members is afforded by the opinion expressed at Islington on October 31, on the occasion of the formation of a non-panel society, when the following resolution was carried without a dissentient in a meeting of between thirty and forty practitioners: "This meeting of non-panel practitioners of St. Pancras and Islington expresses its profound dissatisfaction with the manner in which the British Medical Association has persistently ignored the interests of non-panel practitioners."

The doctors throughout the country who have declined service under the Insurance Act may well congratulate themselves on having nothing to do with a system embracing such possibilities as were revealed at a recent meeting of the Worcester City Insurance Committee, when it was actually decided to allow insured persons to make their own arrangements for "treatment" by "herbalists." There is, of course, no novelty in this decision of an Insurance Committee, and Mr. Masterman, to his disgrace, refused months ago in the House of Commons to disallow the competency of "herbalists" to give medical certificates for Insurance Act purposes; each Committee, he said, would decide what evidence of sickness it deemed sufficient. It is to be noted that when "contracting-out" is allowed the attendance must, by the regulations, be of a quality not inferior to that given by panel doctors. So that the herbalist is now raised to the status of a qualified practitioner. It remains to be seen what substitute the Commissioners will find for the death certificates, which in the present state of the law may only be furnished by registered practitioners. No doubt as their regulations have the force of an Act of Parliament the Commissioners will be equal to the occasion and will make an order for the inclusion of "herbalists" in future issues of the Medical Register, and will further instruct our Universities to lose no time in conferring honorary medical degrees on herbalists and other quacks wishing to treat insured persons, in order to facilitate the furnishing of certificates and to enable medical evidence to be given at coroners' inquests and in the law courts. But, in all seriousness, we may well ask, Are the panel doctors going to be content to degrade their calling by throwing in their lot with herbalists, Christian Scientists, and the like, and so continue in support of a scheme where the terms of service are now indeed seen to be derogatory? If they are, goodbye to the last thread of the bond of union with the non-panel section of the profession. The Insurance Committees are, as we now see, free to grant facilities for the "treatment" of insured persons by quacks, but not by non-panel registered practitioners. The latter are to stand aside and see their former patients taken from them and practically invited to become the prey of some herbalist or other quack desirous of "attending" insured persons.

### Herbalists and Insured Persons.

The quacks are adjudged worthy to be entrusted with the lives of the insured, while the non-panel doctors are refused the same right of contracting-out. This is a state of affairs directly sanctioned by responsible Ministers of the Crown. It is difficult to speak or write in restrained language of such a disgraceful condition of things, and it is doubtful if one's uppermost feeling in the matter is contempt and indignation for the methods of administration of the Act, or astonishment and despair at the ignorance and apathy of the public which make such things possible in this twentieth century. It appears that at the Worcester Committee meeting Drs. Neville Crowe and S. Wellesley Coombs attempted to induce the Committee to see the outrageous character of the proposal to allow insured persons to receive treatment from any other than qualified medical practitioners, but without effect. The members present decided against the doctors' protest by fifteen to nine, the minority of nine including the four medical members. Surely it is degrading to the dignity of professional men to waste time in discussing affairs with a body so oblivious of the preposterous character of its findings as the Worcester Committee is shown to be. This matter of the relation of herbalists to the Insurance Act deserves full publicity, and it is to be hoped that some at least of the community will realise the nature of the channels into which the public money is being diverted in the administration of the Act.

Reference was made last week in these columns to the valuable work of the Special Commissioner of the *Lancet* in connection with Insurance affairs throughout the country, and the excellent reports thereon now appearing in that journal. Some of the reading is of a startling character and deserves publicity. For example, when remembering that the tuberculous insured persons were promised first-class hotel accommodation, one wonders to what sort of hotels the Commissioners and Insurance Committees are accustomed when the *Lancet's* Commissioner can write as follows. He is dealing with the situation as it exists at Newcastle, and in describing the tuberculosis dispensary he says: "It is very accessible, but the street is so noisy that it is difficult to examine the patients. Then the dispensary only consists of two small rooms or compartments. One of these is the clerk's room, the store-room, the writing-room, and the waiting-room, all in one. The other is the consulting-room, the bacteriological laboratory, and the patients' dressing-room, also all in one." (The *Lancet*, October 18, p. 1149.) It is small excuse to say that the present arrangements are but temporary; what one would like to know is how it comes about that premises bearing such a description were selected as being suitable even for a single day. There is gross mismanagement somewhere, and we need something more by way of explanation than that "it appears that when efforts have been made to rent larger and more suitable premises, the people living near have raised objections against so many consumptive patients coming daily into their neighbourhood."

### The Panel Medical Committees.

Mention may be made of the Panel Medical Committees shortly to be elected. According to the Commissioners' regulations three-fourths of those elected must be on the panels, there being no rule as to the remaining fourth. It is to be noted that in the election only panel men may vote, and it is, therefore, impossible that a committee so chosen can be regarded as properly representative of the practitioners of the district. This will be especially so in the London area, having regard to the figures given below concerning the panels. The restriction of the voting to panel men goes to emphasise the now obvious cleavage of the profession into panel and non-panel sections. Attention may be directed to the fact that in connection with the coming election of Panel Committees, the Commissioners have adopted a peculiar and unexplained hustling procedure very difficult to understand on the assumption that a fair result is desired. Practically no time is given for nominations to be effected, or for a consideration of the regulations respecting the elections. Reference has been made above to the state of the London panels, and as contradictory versions have appeared at various times, the following figures are quoted from the authority of a well-known member of the London Insurance Committee. There are, approximately, 1,500 men on the London panels, 200 of these being residents at hospitals and other institutions. The remaining 1,300 are panel doctors engaged in working the Act as general practitioners. There are in all 6,200 medical men in the London area. At a liberal estimate, we may class 2,200 of these as consultants, retired, and institutional men who are thus ineligible for panel work. This leaves 4,000 general practitioners eligible for the panels. As we have already seen, only 1,300 of these, or less than one-third, have undertaken the work.

These facts bear closely on the non-panel movement, for they disclose features in the working of the Act which not only justify that movement, but which make it clear that if the medical profession is to regain prestige, effort will only come from a combination of non-panel men. Such combination, it is encouraging to know, is now being effected, and may be expected to receive a powerful impetus at the Conference convened by the National Medical Union which, it is understood, will be held in London at the end of the present month.

